# Non-isoprenoid botryane sesquiterpenoids from basidiomycete *Boletus edulis* and their cytotoxic activity

**DOI:** 10.1007/s13659-011-0005-9

**Published:** 2011-09-02

**Authors:** Tao Feng, Zheng-Hui Li, Ze-Jun Dong, Jia Su, Yan Li, Ji-Kai Liu

**Affiliations:** 1 State Key Laboratory of Phytochemistry and Plant Resources in West China, Kunming Institute of Botany, Chinese Academy of Sciences, Kunming, 650201 China; 2 Graduate School of Chinese Academy of Sciences, Beijing, 100039 China

**Keywords:** botryane, sesquiterpenoid, boledulin, *Boletus edulis*

## Abstract

Three non-isoprenoid botryane sesquiterpenoids, named boledulins A-C (**1–3**), have been isolated from the cultures of basidiomycete *Boletus edulis* Bull. The structures were established by means of spectroscopic methods. Boledulin A (**1**) exhibited moderate inhibitory activity against five human cancer cell lines.

**Electronic Supplementary Material:**

Supplementary material is available for this article at 10.1007/s13659-011-0005-9 and is accessible for authorized users.

## Introduction

Botryane sesquiterpenoids possess a non-isopreniod system skeleton, which have been found limited to several fungi such as *Botrytis cinerea*[1] and *Daldinia concentrica*[2]. The representative botryane sesquiterpenoids are botrydial[1a] and its derivatives, which are characterized from phytopathogenic fungus *B. cinerea*. These sesquiterpenoids showed a wide range of biological activities. For instance, they were responsible for the typical lesions associated with *B. cinerea* infection, and they played an important role in the pathogenicity of the organism *in vivo*.[1c,3] Botryane sesquiterpenoids attracted great interests of chemists to carry out a large number of investigations including chemical transformations,[3b] structure-activity relationships,[1c] synthesis,[4] and biosynthesis[5]. Our group has long been focused on the chemical study on higher fungi. Recently, three new botryane sesquiterpeniods, boledulins A-C (**1**–**3**), have been isolated from cultures of *Boletus edulis* Bull, an edible basidiomycete collected from southwest of China. The structures were established by extensive spectroscopic data. It is noted that compound **3** is a 15-nor-botryane sesquiterpenoid which was seldom found previously. In addition, compounds **1**–**3** were evaluated for their cytotoxicity against five human cancer cell lines. This paper reports the isolation, structural elucidation, and cytotoxicity of these compounds.

## Results and Discussion

Compound **1** was isolated as optical active white solid ($[\alpha]_D^{20}$ + 16.8). HRESIMS displayed an $[M + Na]^+$ peak at *m/z* 337.1990 (calcd 337.1990 for $C_{17}H_{30}O_5Na$) indicating a molecular formula $C_{17}H_{30}O_5$ corresponding to three degrees of unsaturation. IR spectrum revealed the existence of hydroxy and carbonyl groups due to absorption bands at 3439 and 1729 $cm^{-1}$, respectively. The $^{13}C$ NMR spectrum gave 17 carbon resonances (Table 1). Besides two methoxy signals at $\delta_C$ 51.6 and 59.4, 15 resonances can be ascribable to four methyls, three $sp^3$ methylenes, four $sp^3$ methines, three $sp^3$ quaternary carbons, and one $sp^2$ quaternary carbon at $\delta_C$ 174.6. These data suggested that compound **1** might be a bicyclic sesquiterpenoid.

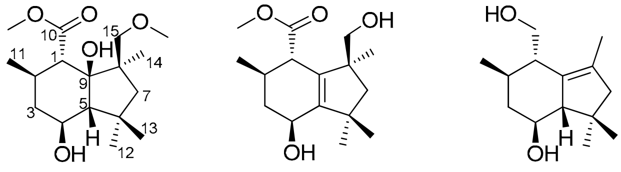

**1** **2** **3**

In the HMBC spectrum, the correlation of a methoxy signal at $\delta_H$ 3.70 (3H, s) with $\delta_C$ 174.6 (s, C-10) established a methyl ester group. A key correlation of $\delta_H$ 2.45 (1H, d, $J$ = 12 Hz, H-1) with C-10 suggested the linkage of C-10 to the methine. Starting from this methine, a structural fragment was established by the analysis of $^1H$-$^1H$ COSY spectrum as shown in Figure 1. The HMBC correlations of H-1 and $\delta_H$ 1.56 (1H, d, $J$ = 11.0 Hz, H-5) with the oxygen-containing quaternary carbon signal at $\delta_C$ 87.8 (s, C-9) revealed the connections of C-9 to C-1 and C-5, respectively. Hence, ring A was established as shown in Figure 1. Ring B was readily built due to the rest of carbon and proton resonances leading to the only perfect linkage, which was supported further by the HMBC correlations (Figure 1). In addition, a methoxy group placed at

*To whom correspondence should be addressed. E-mail: jkliu@mail.kib.ac.cn.

C-15 was also deduced from the HMBC correlation (Figure 1).

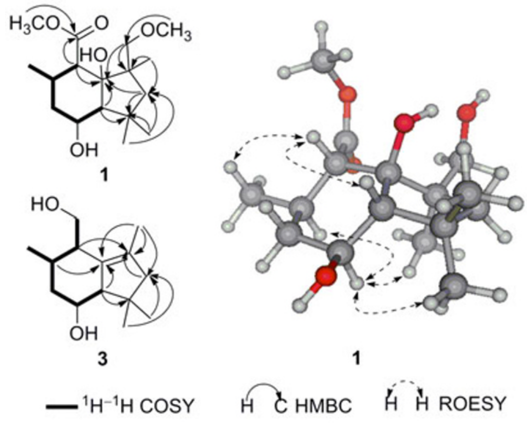

**Figure 1.** Selected 2D NMR correlations of **1** and **3**.

In the ROESY spectrum, the observed cross peaks of H-1/H-5, H-2/H-4, H-1/$CH_3$-11, H-4/$CH_3$-12 and H-4/$CH_3$-14 suggested H-1, H-5, and Me-11 in the same side, while H-2, H-4, Me-12, and Me-14 in the opposite side. Further, these above ROESY cross peaks limited OH-9 to be the same side with H-1. Structurally, compound **1** should be a derivative of botrydial ($[\alpha]^{20}_{D}$ + 34),[1a] the first botryane sesquiterpenoid isolated from *Botrytis cinerea*,[1a] whose absolute configuration has been identified on the basis of synthesis and biosynthesis[4-6].

**Table 1. $^{13}$C NMR data for boledulins A–C (1–3).**

| position | **1**[a] | **2**[b] | **3**[b] |
|---|---|---|---|
| 1 | 61.3, CH | 44.2, CH | 48.4, CH |
| 2 | 29.9, CH | 33.1, CH | 31.7, CH |
| 3 | 43.6, $CH_2$ | 37.9, $CH_2$ | 44.8, $CH_2$ |
| 4 | 70.2, CH | 67.3, CH | 70.4, CH |
| 5 | 68.4, CH | 150.9, C | 65.8, CH |
| 6 | 36.7, qC | 44.9, qC | 37.7, qC |
| 7 | 53.0, $CH_2$ | 51.5, $CH_2$ | 56.6, $CH_2$ |
| 8 | 49.4, qC | 50.3, qC | 129.4, qC |
| 9 | 87.8, qC | 136.4, qC | 131.4, qC |
| 10 | 174.6, qC | 175.3, qC | 60.5, $CH_2$ |
| 11 | 21.1, $CH_3$ | 19.4, $CH_3$ | 20.0, $CH_3$ |
| 12 | 28.3, $CH_3$ | 30.1, $CH_3$ | 24.8, $CH_3$ |
| 13 | 36.3, $CH_3$ | 30.6, $CH_3$ | 31.2, $CH_3$ |
| 14 | 21.8, $CH_3$ | 23.6, $CH_3$ | 15.0, $CH_3$ |
| 15 | 78.0, $CH_2$ | 68.6, $CH_2$ | |
| COOC$H_3$ | 51.6, $CH_3$ | 52.3, $CH_3$ | |
| OC$H_3$ | 59.4, $CH_3$ | | |

[a]Measured in $CDCl_3$ at 100 MHz;
[b]Measured in $CDCl_3$ at 150 MHz.

Accordingly, the absolute configuration of **1** could be determined as 1*S*, 2*R*, 4*S*, 5*R*, 8*S*, 9*S*. Therefore, the structure of **1** (boledulin A) was established.

Compound **2** was isolated as a colorless oil, that gave an [M + Na]$^+$ peak at *m/z* 305.1720 (calcd for $C_{16}H_{26}O_4Na$, 305.1728) in the positive ion HRESIMS, consistent with the molecular formula $C_{16}H_{26}O_4$ indicating four degrees of unsaturation. The 1D NMR spectroscopic data (Tables 1 and 2) suggested that the backbone of **2** was the same as that of **1**. Differences between them were identified to be a new double bond ($\delta_C$ 136.4 and 150.9) and the loss of a methoxy group at C-15 in **2**. The HMBC correlations of $\delta_H$ 0.93 (3H, s, Me-14) with $\delta_C$ 136.9 (s, C-9) and $\delta_H$ 1.16 and 1.34 (each 3H, s, Me-12 and Me-13, respectively) with $\delta_C$ 150.9 (s, C-5) suggested the double bond placed between C-5 and C-9. The HMBC correlations between H-1, H-4 and C-5, C-9 were also observed. Detailed analysis of other 2D NMR data confirmed that the other parts of **2** were the same as those of **1**. Therefore, the structure of **2** (boledulin B) was established.

Compound **3** was isolated as white solid. The molecular formula $C_{14}H_{24}O_2$ was established by HREIMS at *m/z* 224.1765 [M]$^+$ (calcd for $C_{14}H_{24}O_2$ at *m/z* 244.1776 [M]$^+$) indicating three degrees of unsaturation. The $^1$H NMR spectrum displayed similar patterns to those of **1** and **2** including clear signals for four methyl signals (three singlets and one doublet) (Table 2). The $^{13}$C NMR spectrum revealed 14 carbon resonances ascribable to four $sp^3$ methyls, three $sp^3$ methylenes, four $sp^3$ methines, one $sp^3$ quaternary carbon, and two $sp^2$ quaternary carbons (Table 1). These data suggested that compound **3** might be a bicyclic nor-sesquiterpenoid.

**Table 2. $^1$H NMR Data for Boledulins A-C (1-3).**

| position | **1**[a] | **2**[b] | **3**[b] |
|---|---|---|---|
| 1 | 2.45, d (12.0) | 2.97, d (6.0) | 1.83, m |
| 2 | 1.92, m | 1.89, m | 1.60, m |
| 3a | 1.08, d (12.0) | 1.58, m | 1.20, m |
| 3b | 1.95, m | 1.92, m | 1.92, m |
| 4 | 3.92, ddd (11.0, 4.9, 4.6) | 4.32, dd (9.5, 6.6) | 3.56, ddd (10.4, 6.4, 2.0) |
| 5 | 1.56, d (11.0) | | 2.04, d (6.4) |
| 7 | 1.15, d (12.8) | 1.49, d (13.2) | 2.05, d (16.8) |
| | 2.42, d (12.8) | 1.98, d (13.2) | 2.19, d (16.8) |
| 10 | | | 3.81, dd (11.6, 4.8) |
| | | | 4.07, dd (11.6, 3.3) |
| 11 | 0.87, d (6.6) | 0.95, d (6.6) | 1.00, d (6.4) |
| 12 | 1.28, s | 1.16, s | 1.08, s |
| 13 | 1.26, s | 1.34, s | 1.17, s |
| 14 | 0.97, s | 0.93, s | 1.78, s |
| 15a | 3.01, d (10.2) | 3.07, d (10.4) | |
| 15b | 3.31, d (10.2) | 3.09, d (10.4) | |
| COOC$H_3$ | 3.70, s | 3.69, s | |
| OC$H_3$ | 3.27, s | | |

[a]Recorded in $CDCl_3$ at 400 MHz; [b]Recorded in $CDCl_3$ at 600 MHz.

The $^{13}$C NMR signal at $\delta_C$ 60.5 (t, C-10) allowed the existence of an oxygen-containing methylene, and starting from which, two structural fragments of $CH_3CH$- and $CH_2CHCHCH_2CHCH$- were established by the $^1$H–$^1$H COSY spectrum as shown in Figure 1. In the HMBC spectrum, the key correlations of $\delta_H$ 1.83 (1H, m, H-1) with $\delta_C$ 131.4 (s, C-9) and $\delta_H$ 2.04 (1H, d, $J$ = 6.4 Hz, H-5) with $\delta_C$ 131.4 (s, C-9) suggested the link of C-9 to C-1 and C-5, respectively. Hence, the structure of ring A was established. The HMBC correlation of $\delta_H$ 2.05 and 2.19 (each 1H, d, $J$ = 16.8 Hz, H-7a and H-7b, respectively) with $\delta_C$ 129.4 (s, C-8) suggested a double bond between C-8 and C-9, which was derived from the degradation of a carbon at C-8. Analysis of other 1D and 2D NMR data established ring B as depicted in Figure 1. Therefore, compound **3** was established to be a 15-nor-botryane sesquiterpenoid and named as boledulin C.

**Table 3. Cytotoxicity for Boledulins A-C (1-3) ($IC_{50}$, $\mu$M).**

| Compd. | HL-60 | SMMC-7721 | A-549 | MCF-7 | SW480 |
|---|---|---|---|---|---|
| **1** | 2.6 | 8.4 | 8.3 | 3.4 | 3.5 |
| **2** | > 40 | > 40 | > 40 | > 40 | > 40 |
| **3** | > 40 | > 40 | > 40 | > 40 | > 40 |
| cisplatin | 1.1 | 14.5 | 12.8 | 13.0 | 12.6 |
| taxol | < 0.008 | < 0.008 | < 0.008 | < 0.008 | < 0.008 |

All compounds were evaluated for their cytotoxicities against five human cancer cell lines using the MTT method as reported previously.[7] The result displayed that compound **1** showed moderate cytotoxicity against five human cancer cell lines using cisplatin as the positive control, while compounds **2**–**3** were inactive against all the tested cell lines with $IC_{50}$ values of more than 40 $\mu$M (Table 3).

## Experimental Section

**General Experimental Procedures.** Optical rotations were measured with a Jasco P-1020 polarimeter. IR spectra were obtained on a Bruker FT-IR Tensor 27 spectrometer using KBr pellets. 1D and 2D NMR spectra were run on an AV-400 MHz or a Bruker avance III-600 MHz spectrometer with TMS as an internal standard. Chemical shifts ($\delta$) were expressed in ppm with reference to solvent signals. HREIMS were recorded on a Waters Auto Premier P776 spectrometer. HRESIMS were recorded on an API QSTAR Pulsar i spectrometer. Column chromatography (CC) was performed on silica gel (200-300 mesh, Qingdao Marine Chemical Ltd., Qingdao, People's Republic of China). An Agilent 1100 series instrument equipped with Agilent ZORBAX SB-C18 column (5 $\mu$m, 4.6 mm × 150 mm) was used for high-performance liquid chromatography (HPLC) analysis, and a semipreparative Agilent ZORBAX SB-C18 column (5 $\mu$m, 9.4 mm × 150 mm) was used for the sample preparation. Fractions were monitored by TLC (GF 254, Qingdao Haiyang Chemical Co., Ltd. Qingdao), and spots were visualized by 10% $H_2SO_4$ in ethanol.

**Fungal Material and Cultivation Conditions.** The fungi *Boletus edulis* Bull. were collected from Ailao Mountain, Yunnan province, China. A voucher specimen was deposited at State Key Laboratory of Phytochemistry and Plant Resources in West China, Kunming Institute of Botany, Chinese Academy of Sciences. The mycelial cultures were derived from tissue plugs. Culture PDA medium: potato (peeled), 200 g, glucose, 20 g, $KH_2PO_4$, 3 g, $MgSO_4$, 1.5 g, citric acid, 0.1 g, and thiamin hydrochloride, 10 mg, in 1 L of deionized $H_2O$. The pH was adjusted to 6.5 before autoclaving, and the fermentation was carried out on a shaker at 25 °C and 150 rpm for 20 days.

**Extraction and Isolation.** The culture broth (20 L) was extracted three times with EtOAc. The EtOAc lay was evaporated *in vacuo* to yield an extract (8.6 g). The latter was subjected to a silica gel column eluted with petroleum ether-acetone (1:0 to 0:1) to afford fractions1–5. Fraction 2 (1.8 g) was separated by silica gel CC (petroleum ether-$Me_2CO$, 10:1 → 3:1) to afford two subfractions a and b. Fraction a (100 mg) was separated repeatedly by silica gel CC (petroleum ether-EtOAc, 7:1) to afford **3** (10.5 mg). Fraction b (30 mg) was separated further by HPLC (acetonitrile-$H_2O$, 40:60 to 60:40) to yield **1** (2.2 mg) and **2** (1.1 mg).

**Boledulin A (1)**: white solid; $[\alpha]^{20}_D$ + 16.8 ($c$ 0.19, $CHCl_3$); IR (KBr) $\nu_{max}$ 3439, 2924, 2855, 1729, 1629, 1177, 1088 $cm^{-1}$; $^{13}C$ (150 MHz) and $^1H$ NMR (600 MHz) data ($CDCl_3$), see Tables 1 and 2, respectively; positive ion HRESIMS *m/z* 337.1990 (calcd for $C_{17}H_{30}O_5Na$ $[M + Na]^+$, 337.1990).

**Boledulin B (2)**: colorless oil; $[\alpha]^{20}_D$ + 10.3 ($c$ 0.17, $CHCl_3$); $^{13}C$ (150 MHz) and $^1H$ NMR (600 MHz) data ($CDCl_3$), see Tables 1 and 2, respectively; positive ion HRESIMS *m/z* 305.1720 (calcd for $C_{16}H_{26}O_4Na$ $[M + Na]^+$, 305.1728).

**Boledulin C (3)**: white solid; $[\alpha]^{20}_D$ – 1.0 ($c$ 0.18, $CHCl_3$); IR (KBr) $\nu_{max}$ 3251, 2977, 2828, 1699, 1442, 1361, 1037 $cm^{-1}$; $^{13}C$ (100 MHz) and $^1H$ NMR (400 MHz) data ($CDCl_3$), see Tables 1 and 2, respectively; HREIMS *m/z* 224.1765 (calcd for $C_{14}H_{24}O_2$ $[M]^+$, 224.1776).

**Cytotoxicity Assay.** Five human cancer cell lines, breast cancer MCF-7, hepatocellular carcinoma SMMC-7721, human myeloid leukemia HL-60, colon cancer SW480, and lung cancer A-549 cells, were used in the cytotoxic assay. All the cells were cultured in RPMI-1640 or DMEM medium (Hyclone, USA), supplemented with 10% fetal bovine serum (Hyclone, USA) in 5% $CO_2$ at 37 °C. The cytotoxicity assay was performed according to the MTT (3-(4,5-dimethylthiazol-2-yl)-2,5-diphenyl tetrazolium bromide) method in 96-well microplates.[7] Briefly, 100 $\mu$L adherent cells were seeded into each well of 96-well cell culture plates and allowed to adhere for 12 h before drug addition, while suspended cells were seeded just before drug addition with initial density of $1 \times 10^5$ cells/mL. Each tumor cell line was exposed to the test compound dissolved in DMSO at concentrations of 0.0625, 0.32, 1.6, 8, and 40 $\mu$mol in triplicates for 48 h, with cisplatin (Sigma, USA) and taxol (National Institute for the Control of Pharmaceutical and Biological Products, P. R. China) as positive controls. After compound treatment, cell viability was detected and a cell growth curve was graphed. $IC_{50}$ values were calculated by Reed and Muench's method.[8]

## Electronic Supplementary Material

Supplementary material is available in the online version of this article at http://dx.doi.org/10.1007/s13659-011-0005-9 and is accessible for authorized users.

**Acknowledgments**

This work was supported in part by the National Natural Sci This project was supported by the National Basic Research Program of China (973 Program, 2009CB522300), the National Natural Sciences Foundation of China (30830113), and MOST (2009ZX09501-029).

**Open Access** This article is distributed under the terms of the Creative Commons Attribution License which permits any use, distribution, and reproduction in any medium, provided the original author(s) and source are credited.

## Electronic supplementary material

Supplementary material, approximately 438 KB.
