# Supplementary material for: Non-isoprenoid botryane sesquiterpenoids from basidiomycete Boletus edulis and their cytotoxic activity
Source: Nat Prod Bioprospect. 2011 Sep 2;1(1):29–32. doi: 10.1007/s13659-011-0005-9 (PMC4131704; doi:10.1007/s13659-011-0005-9)
Supplement: Supplementary file 1 — Supplementary material, approximately 438 KB. [file 13659_2011_5_MOESM1_ESM.pdf]

Electronic Supplementary Material

Non-isoprenoid botryane sesquiterpenoids from basidiomycete *Boletus edulis* and their cytotoxic activity

Tao FENG,[a] Zheng-Hui LI,[a] Ze-Jun DONG,[a] Jia SU,[a,b] Yan LI,[a] and Ji-Kai LIU[a,]*

[a]State Key Laboratory of Phytochemistry and Plant Resources in West China, Kunming Institute of Botany, Chinese Academy of Sciences, Kunming 650201, China

[b]Graduate School of Chinese Academy of Sciences, Beijing 100039, China

Received 29 June 2011; Accepted 21 July 2011

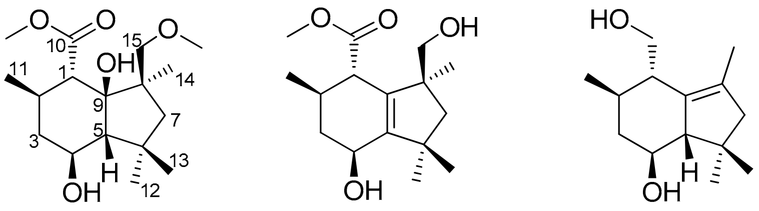

1 **2** **3**

Structures of compounds **1**–**3**.

*To whom correspondence should be addressed. E-mail: jkliu@mail.kib.ac.cn.

Figure 1S-7S. NMR and MS spectra of boledulin A (**1**)

Figure 8S-14S. NMR and MS spectra of boledulin B (**2**)

Figure 15S-21S. NMR and MS spectra of boledulin C (**3**)

Figure 1S. ^{1}H NMR of boledulin A (**1**).

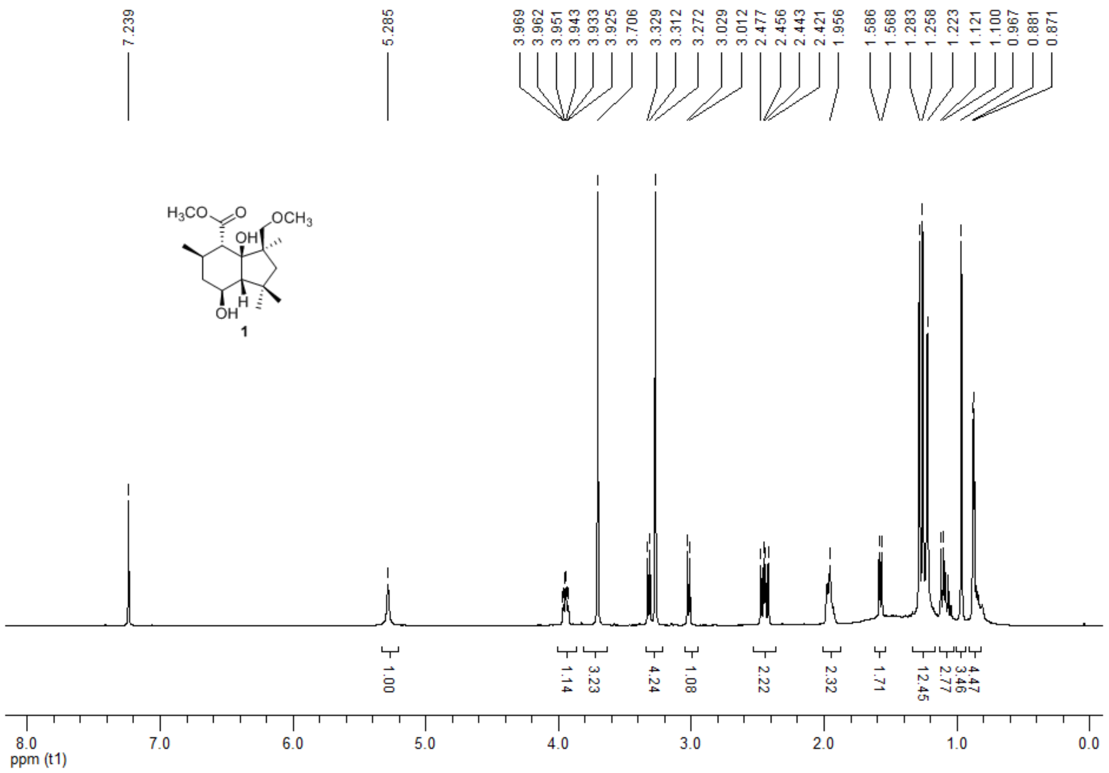

Figure 2S. ^{13}C NMR of boledulin A (**1**).

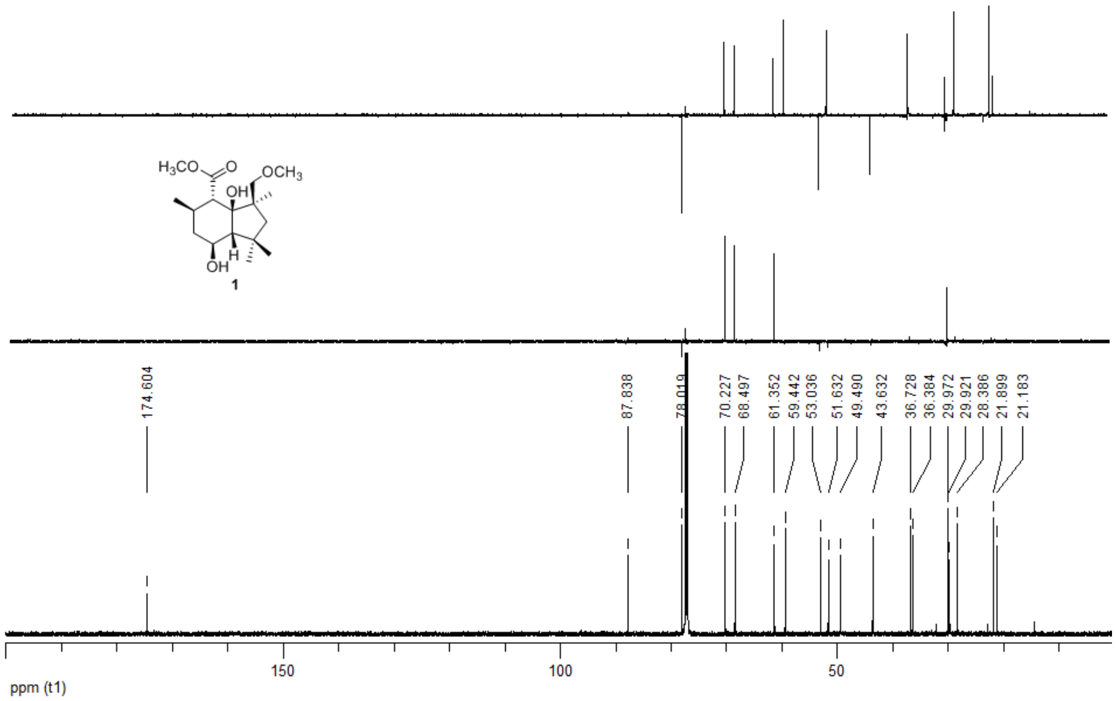

Figure 3S. HSQC of boledulin A (**1**).

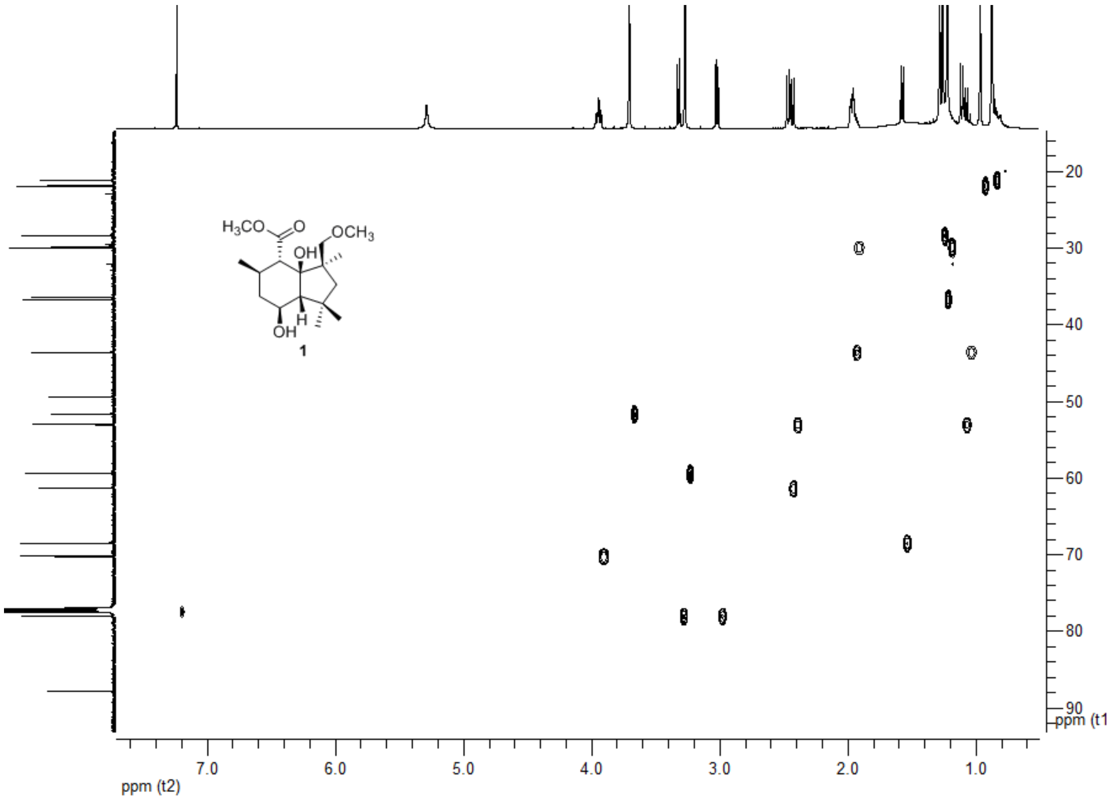

Figure 4S. HMBC of boledulin A (**1**).

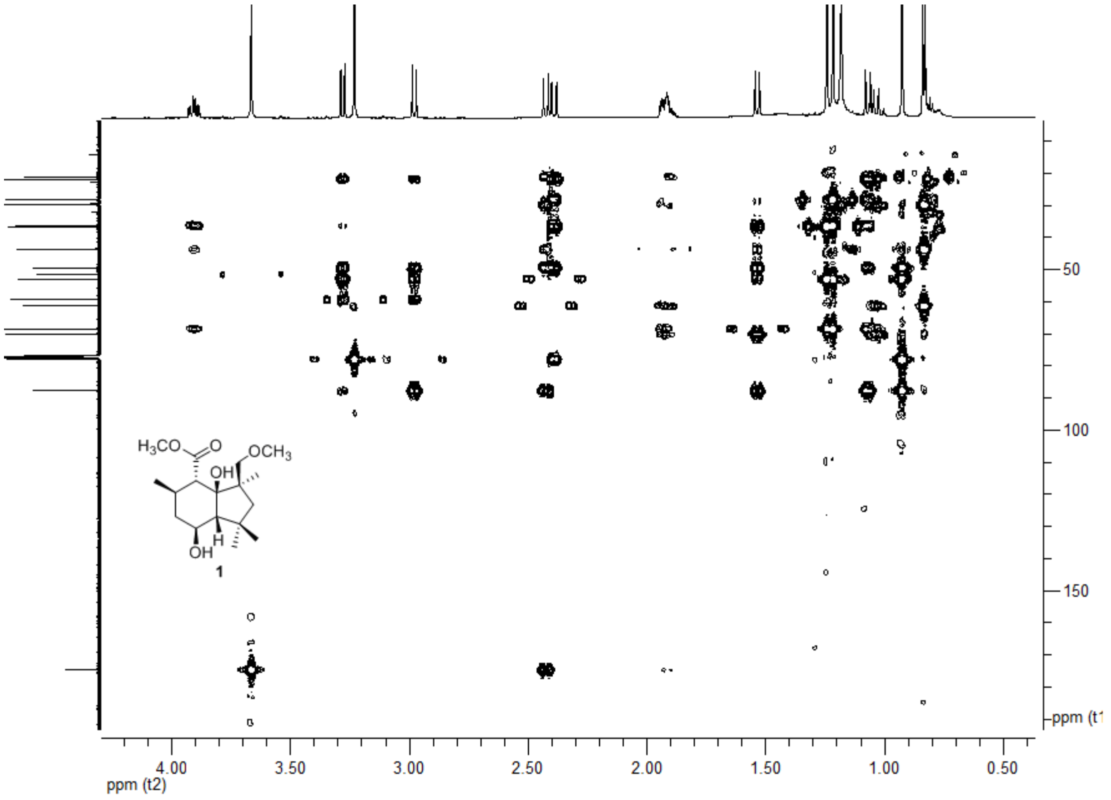

Figure 5S. ^{1}H-^{1}H COSY of boledulin A (**1**).

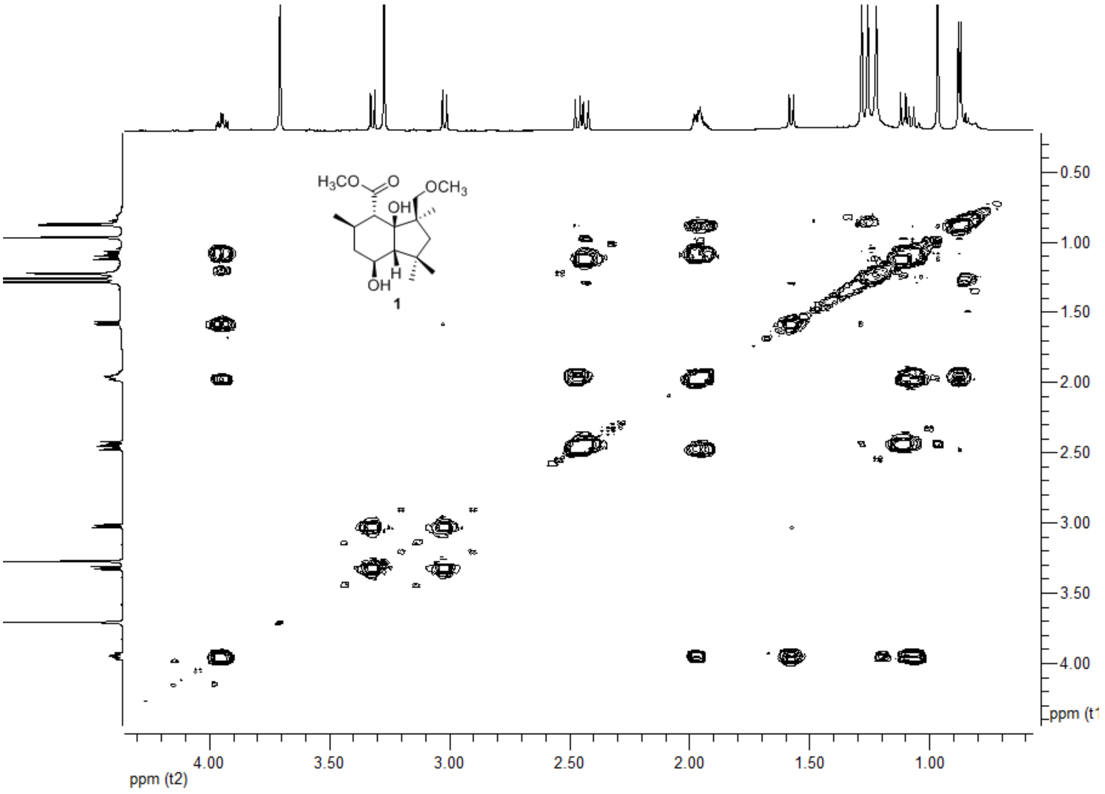

Figure 6S. ROESY of boledulin A (**1**).

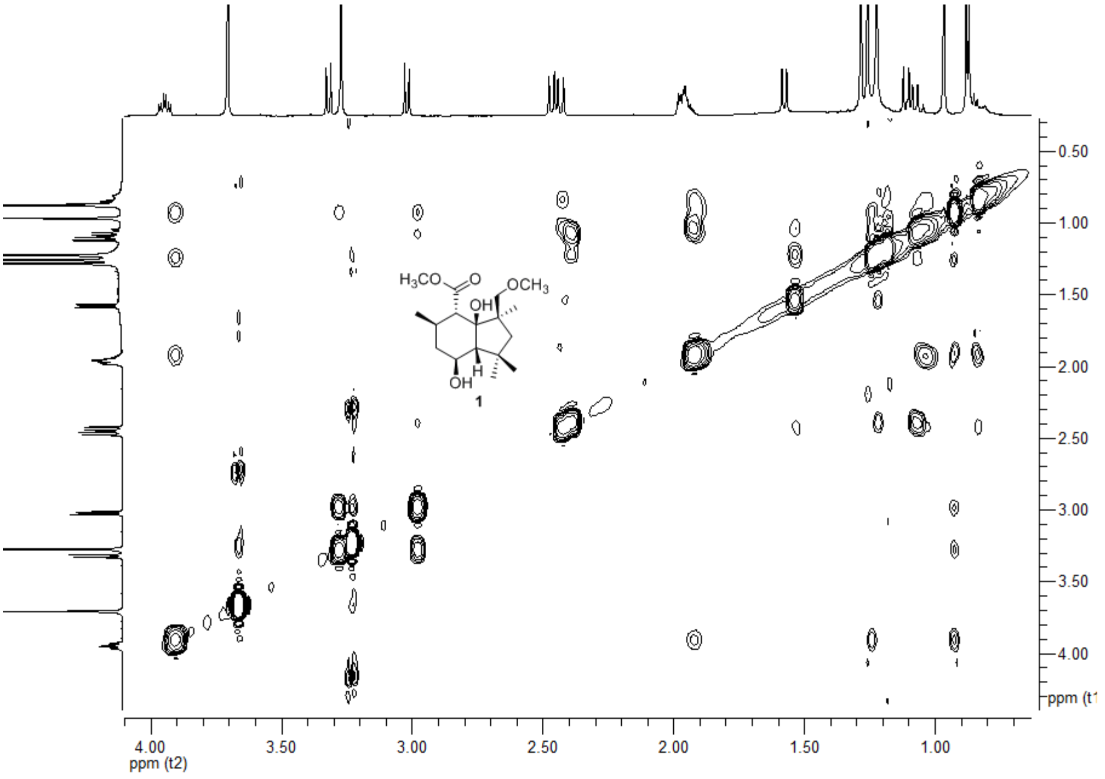

Figure 7S. HRESIMS of boledulin A (**1**).

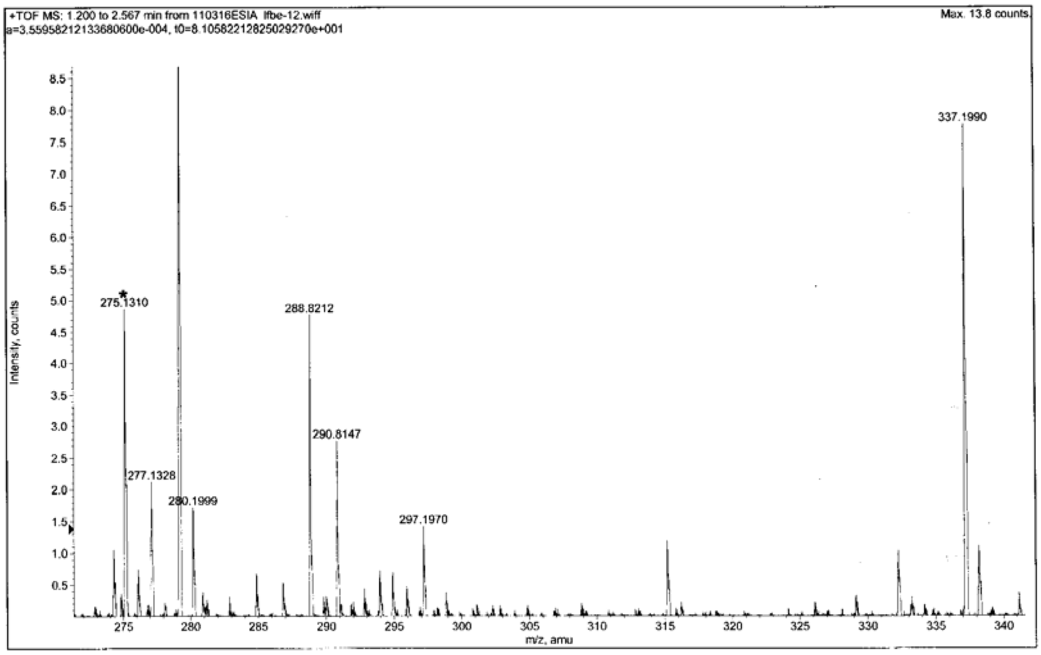

Figure 8S. ^{1}H NMR of boledulin B (**2**).

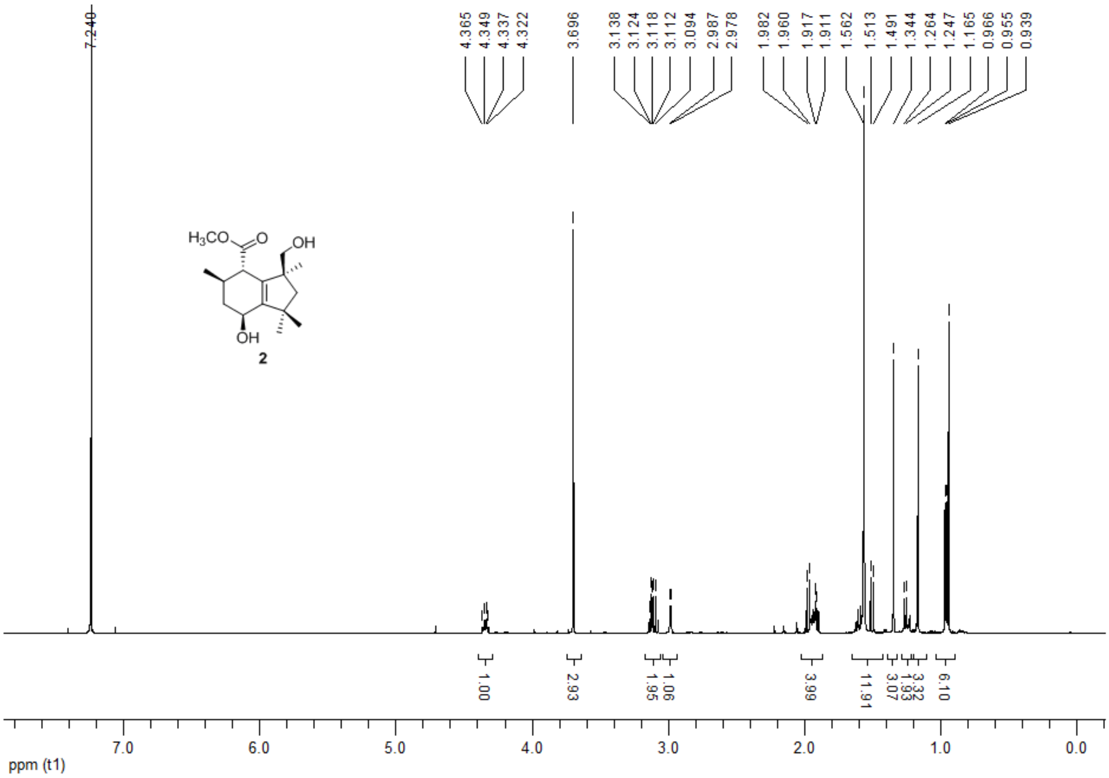

Figure 9S. ^{13}C NMR of boledulin B (**2**).

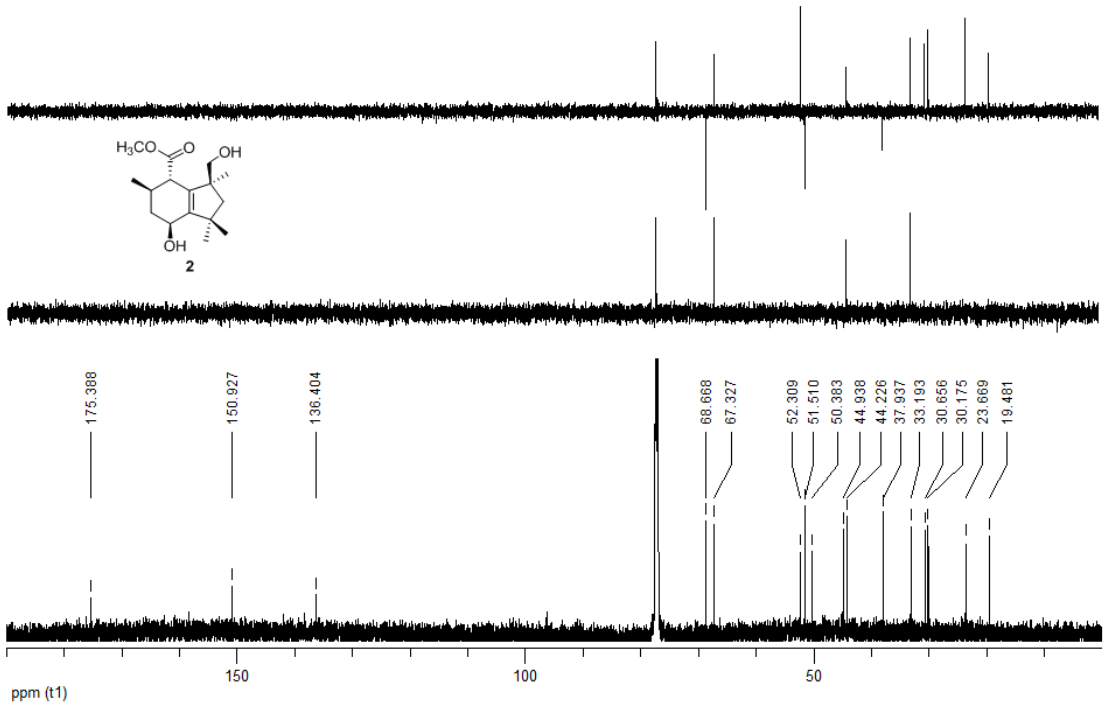

Figure 10S. HSQC of boledulin B (**2**).

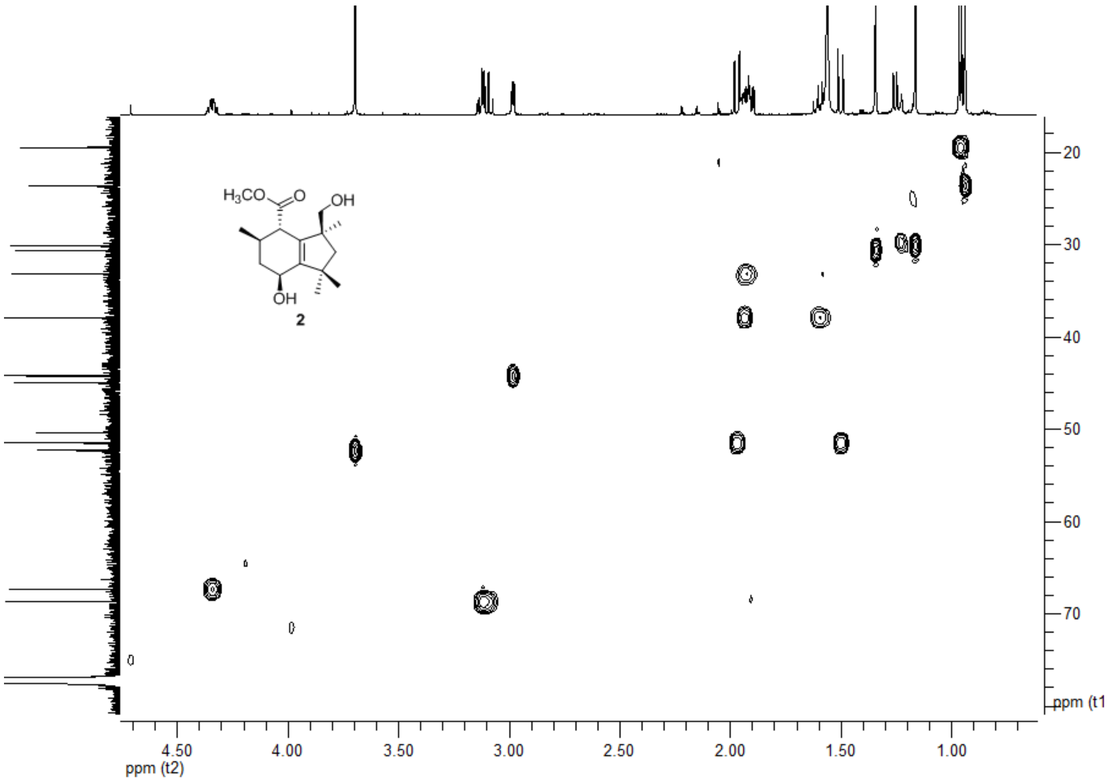

Figure 11S. HMBC of boledulin B (**2**).

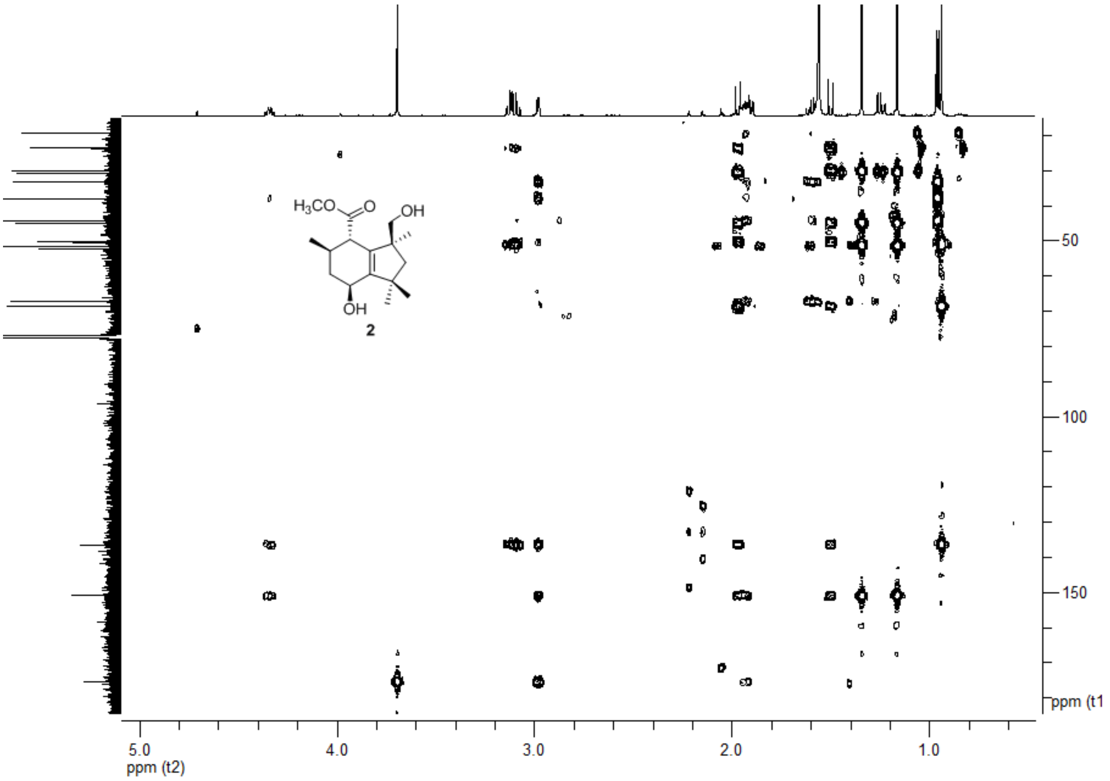

Figure 12S. ^{1}H-^{1}H COSY of boledulin B (**2**).

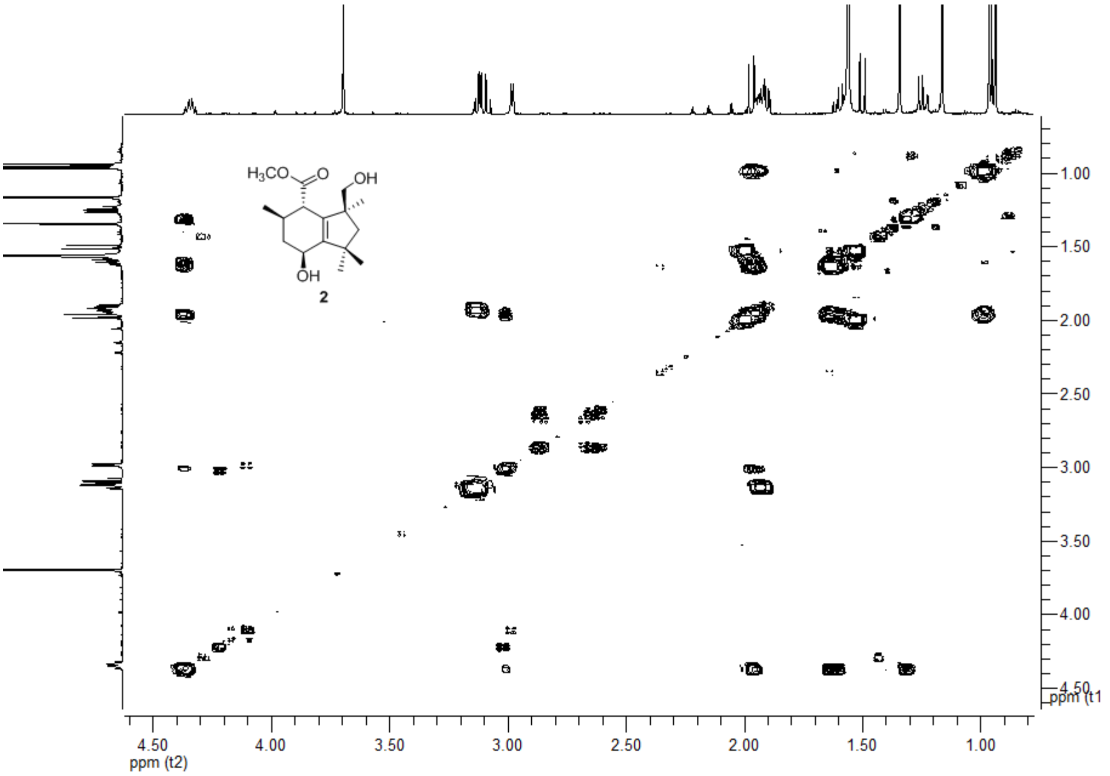

Figure 13S. ROESY of boledulin B (**2**).

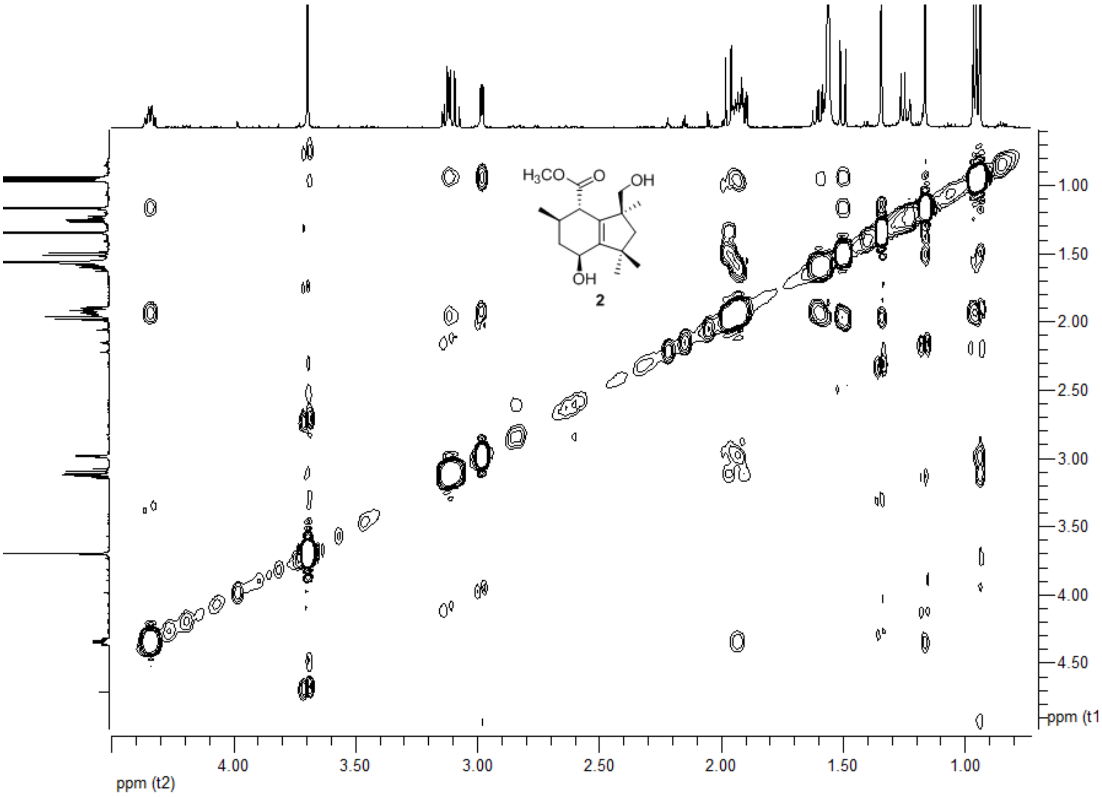

Figure 14S. HRESIMS of boledulin B (**2**).

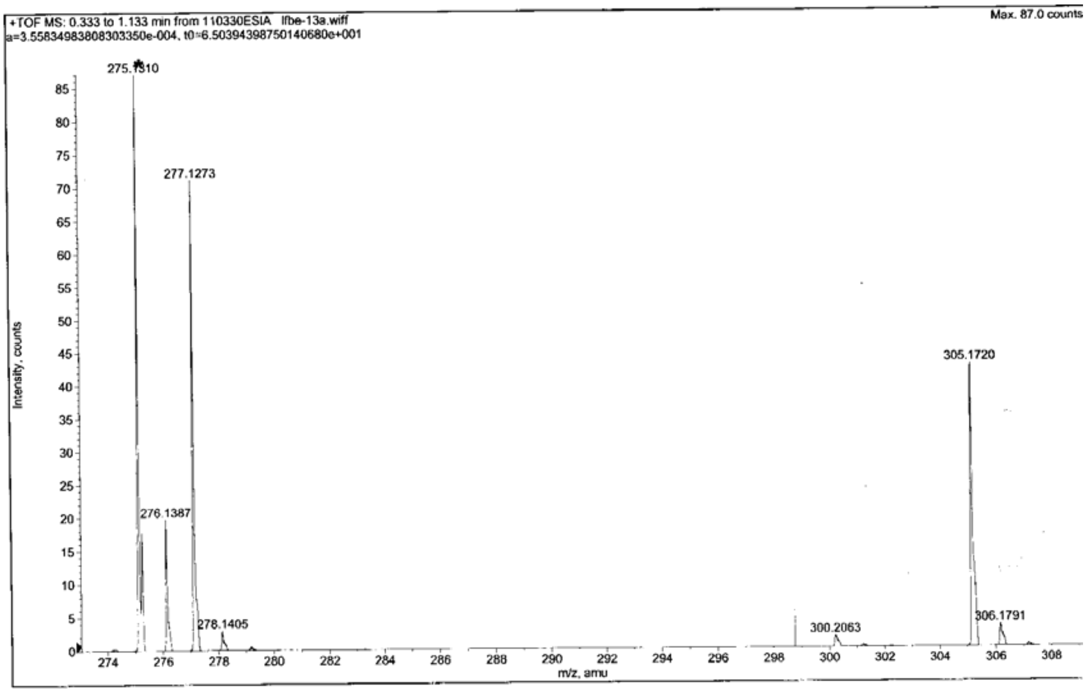

Figure 15S. ^{1}H NMR of boledulin C (**3**).

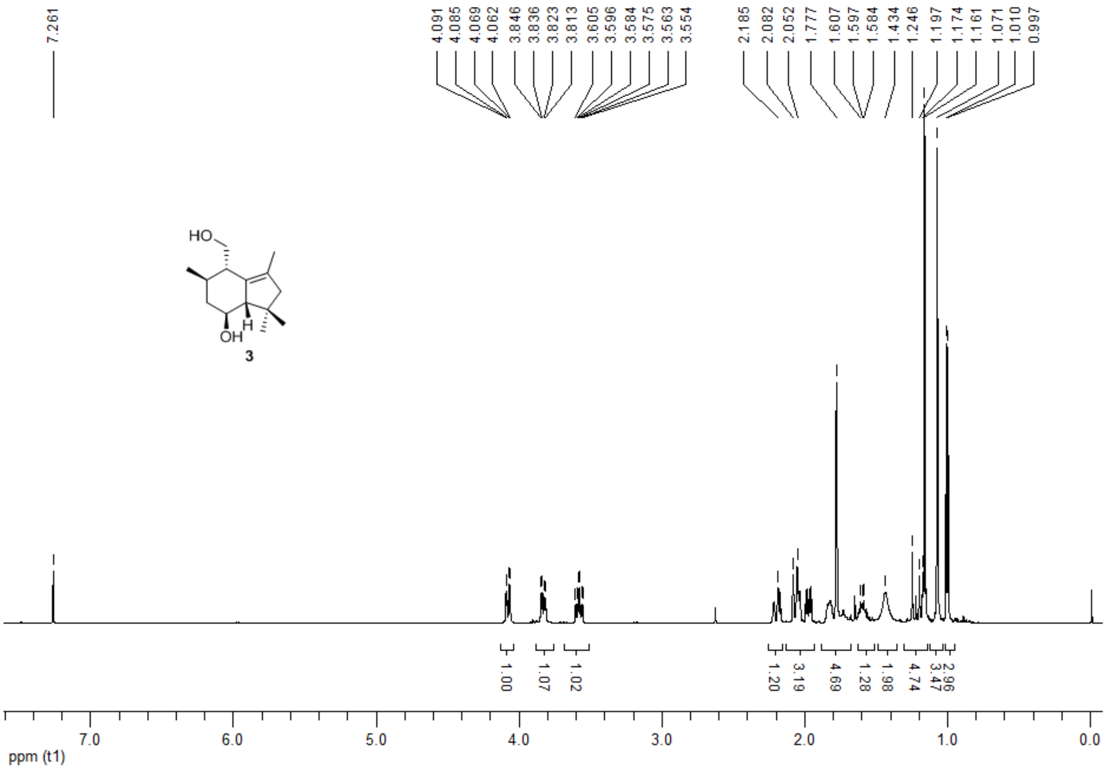

Figure 16S. ^{13}C NMR of boledulin C (**3**).

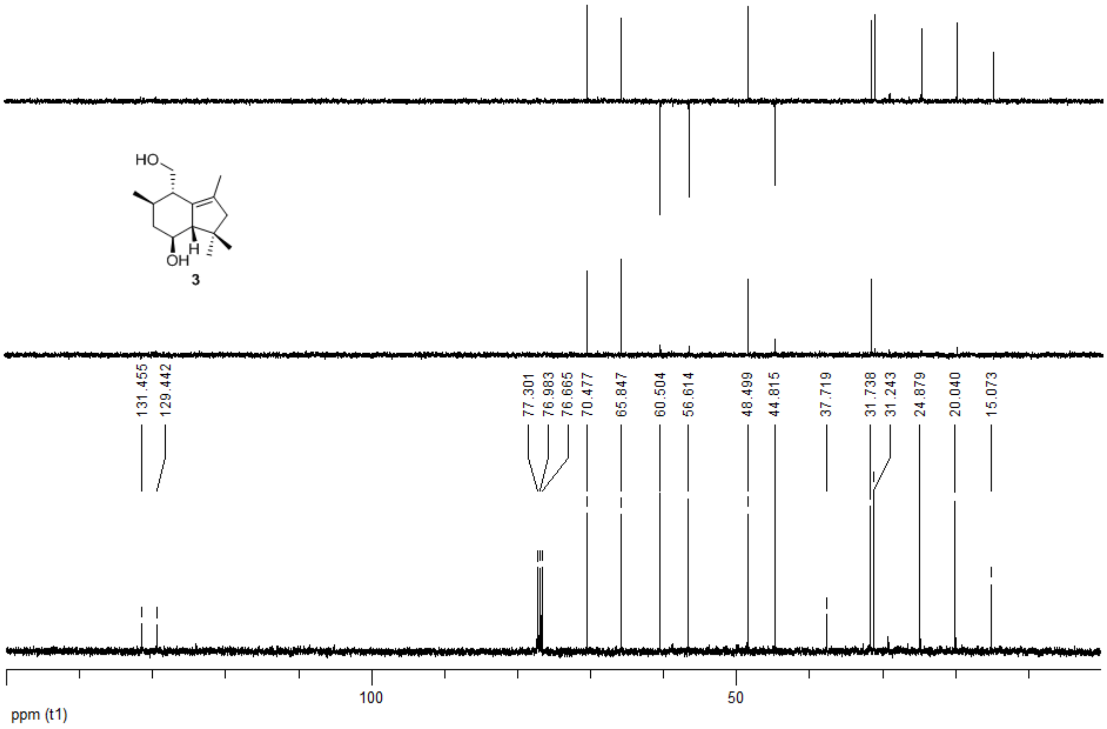

Figure 17S. HSQC of boledulin C (**3**).

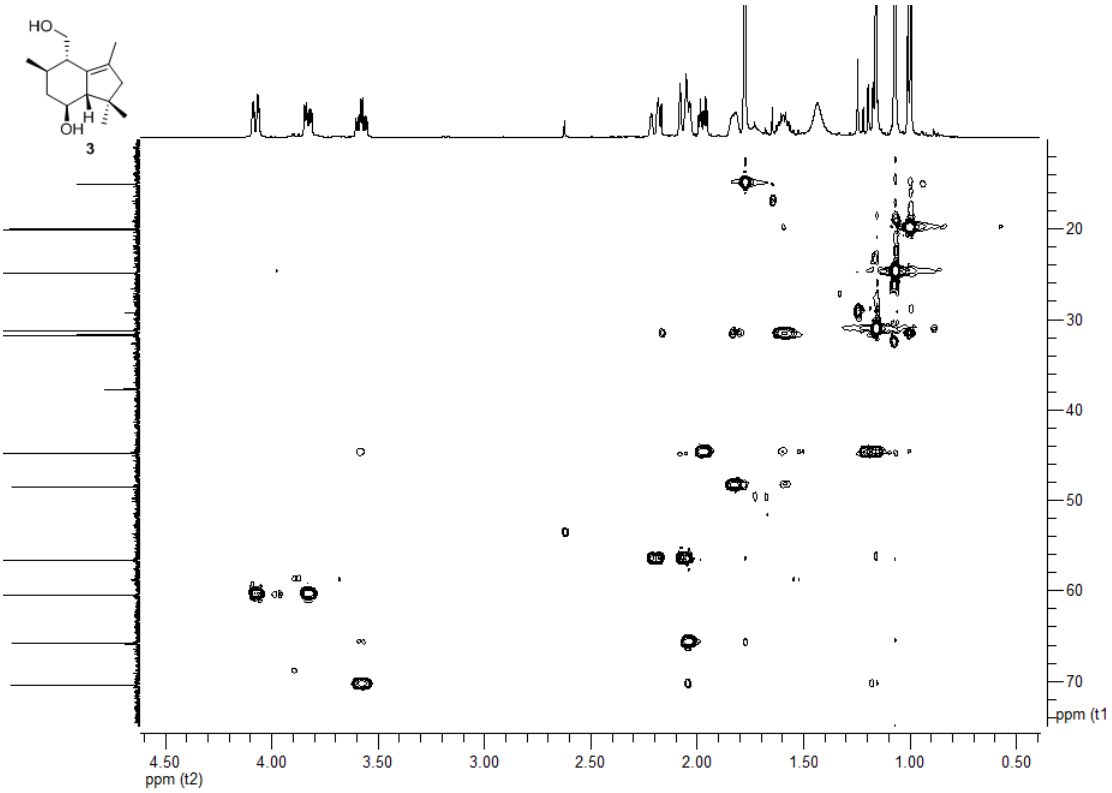

Figure 18S. HMBC of boledulin C (**3**).

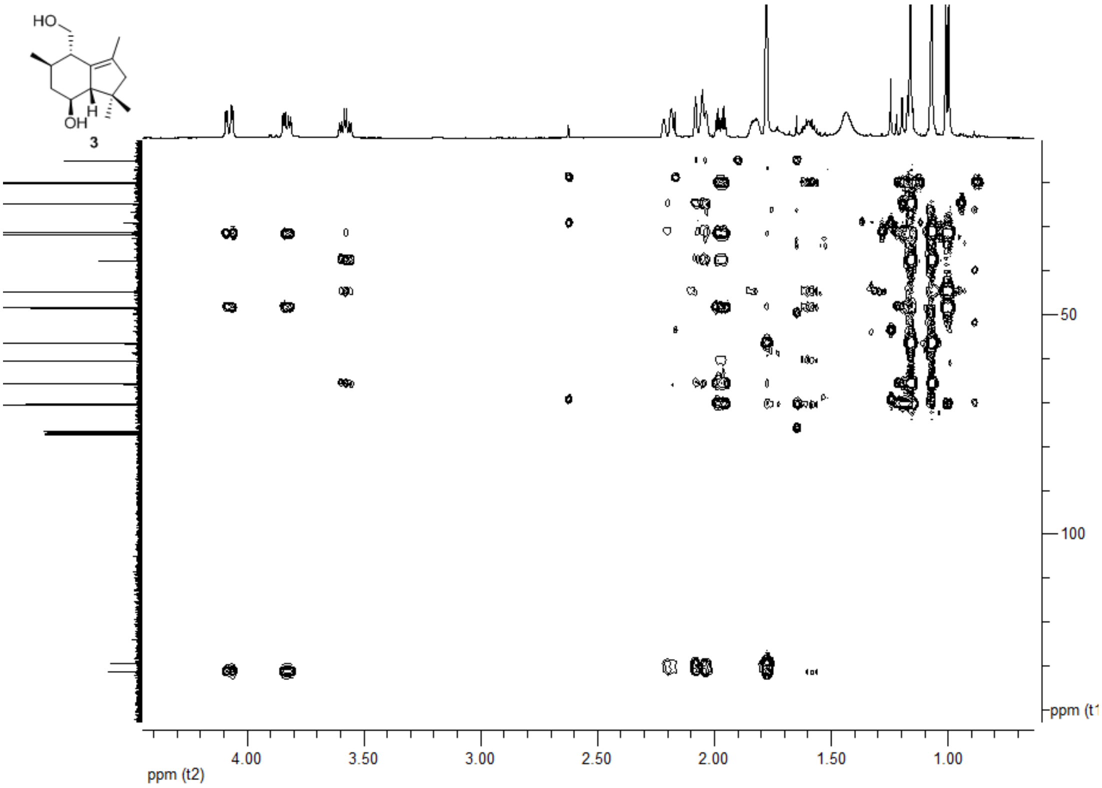

Figure 19S. ^{1}H-^{1}H COSY of boledulin C (**3**).

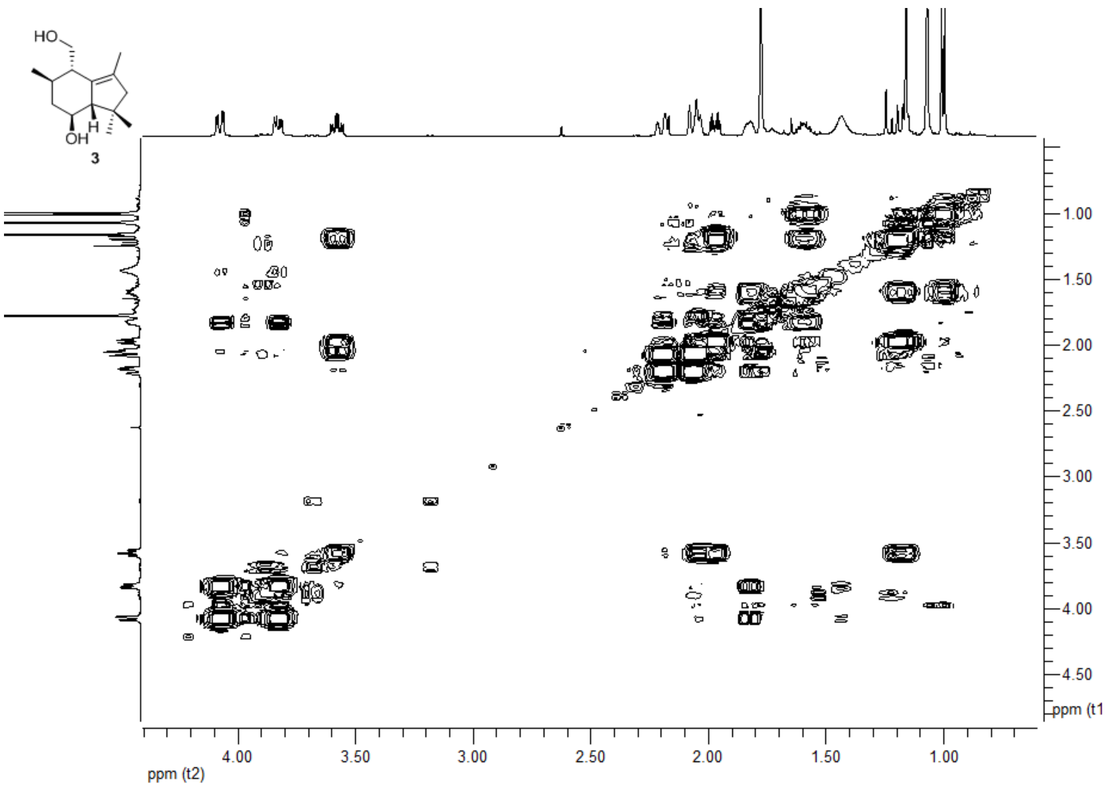

Figure 20S. ROESY of boledulin C (**3**).

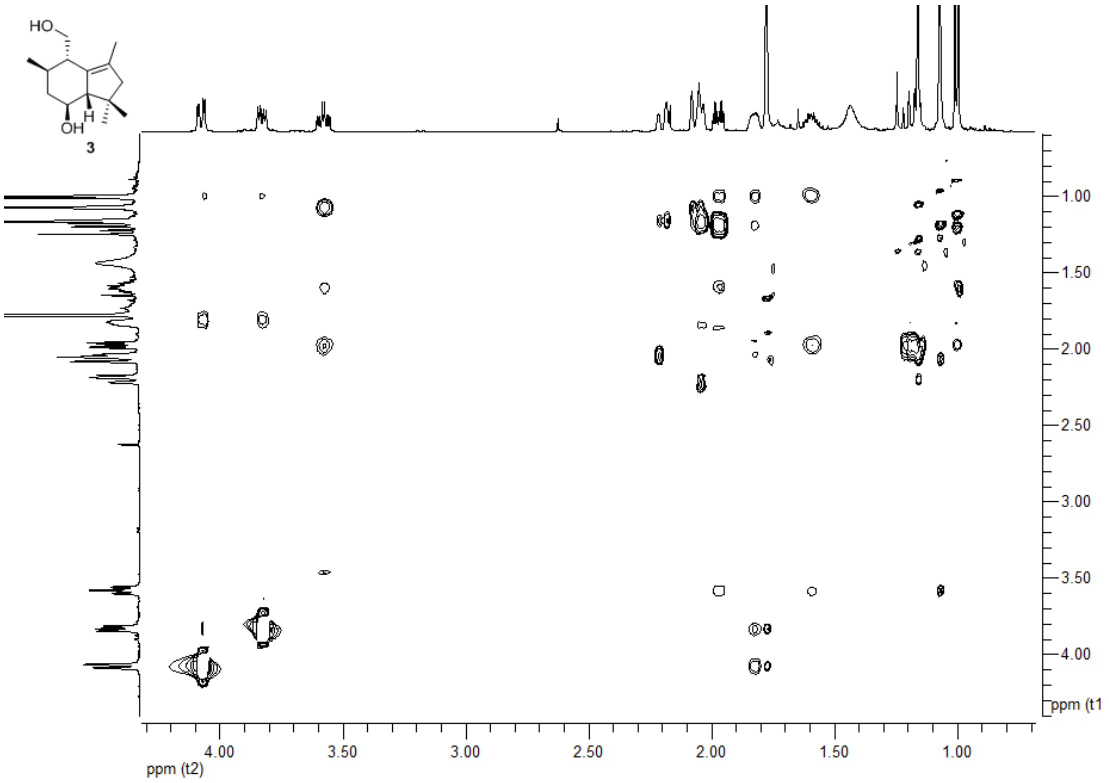

Figure 21S. HREIMS of boledulin C (**3**).

Elemental Composition Report **Page 1**

Single Mass Analysis (displaying only valid results)
Tolerance = 10.0 mDa / DBE: min = 0.5, max = 40.0
Selected filters: None

Monoisotopic Mass, Odd and Even Electron Ions
9 formula(e) evaluated with 1 results within limits (up to 51 closest results for each mass)
Elements Used:
C: 0-200 H: 0-400 O: 0-2

Lfbe-1
17:00:16 22-Jul-2010
Voltage EI+

KIB
M100722EA-02AFAMM 6 (0.551)

Autospec Premier
P776
22.6

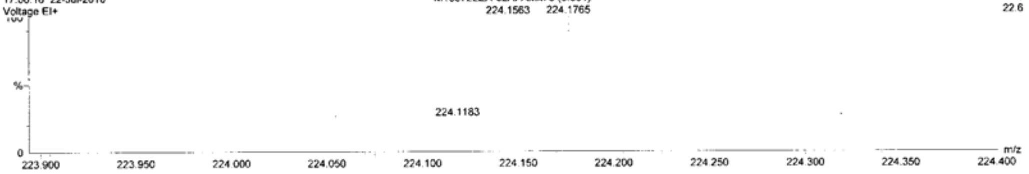

Minimum:				0.5		
Maximum:		10.0	10.0	40.0		
Mass	Calc. Mass	mDa	PPM	DBE	i-FIT	Formula
224.1765	224.1776	-1.1	-4.9	3.0	5546027.5	C14 H24 O2
